# Transcutaneous auricular vagus nerve stimulation (taVNS): a comprehensive review of mechanisms, clinical applications, and future directions

**DOI:** 10.3389/fneur.2026.1933117

**Published:** 2026-09-25

**Authors:** Yun Zhao, Tao Shen, Shushan Jia

**Affiliations:** Department of Anesthesiology, Yantai Affiliated Hospital of Shandong Medical and Pharmaceutical University, Yantai, Shandong, China

**Keywords:** anesthesia, depression, epilepsy, gastrointestinal function, migraine, post-stroke rehabilitation, taVNS

## Abstract

**Purpose:**

Transcutaneous auricular vagus nerve stimulation (Transcutaneous Vagus Nerve Stimulation, TaVNS) is a non-invasive neuromodulation technique that has garnered significant attention. This article aims to summarize its mechanisms of action, clinical applications, and future challenges.

**Methods:**

A non-systematic literature seach was performed in PubMed for articles published. Studies investigating taVNS, gastrointestinal function, anesthesia, depression, epilepsy, migraine, and post-stroke rehabilitation were identified. A total of 72 articles were included in this review after full-text assessment. This work is a narrative review rather than a systematic review. No pre-registered search strategy, formal eligibility criteria, or risk-of-bias assessment was performed. Literature was collected via targeted non-systematic retrieval focusing on preclinical and clinical advances of transcutaneous auricular vagus nerve stimulation. Relevant original articles and reviews were selectively included to summarize current evidence, mechanisms, safety status and future perspectives of taVNS.

**Results:**

The analysis revealed that taVNS delivers stimulation to the auricular branch of the vagus nerve, transmitting signals to the nucleus tractus solitarius (NTS). Subsequently, these signals modulate emotional and cognitive processes via ascending pathways and regulate autonomic nervous system function through descending pathways. A key mechanism involves the activation of the “cholinergic anti-inflammatory pathway,” which suppresses systemic inflammation. Evidence suggested that taVNS holds potential therapeutic value for a variety of conditions, including depression, epilepsy, migraine, and post-stroke rehabilitation.

**Conclusions:**

This review demeonstrates that future research should focus on parameter optimization, mechanistic exploration, and the development of personalized treatment protocols to facilitate its clinical translation.

## 1 Introduction

In recent years, the gut-brain axis, a bidirectional communication system linking the central nervous system and the enteric nervous system, has garnered considerable research attention (1), and its dysfunction is implicated in a variety of gastrointestinal disorders. The bidirectional gut-brain crosstalk under physiological and pathological conditions is primarily mediated through the autonomic nervous system (ANS) and circumventricular organs, among other pathways (2). The ANS consists of the parasympathetic and sympathetic nervous systems, whose pathways encompass the vagus nerve (VN), sacral parasympathetic pelvic nerves, and splanchnic nerves. These mixed nerves facilitate signal transmission between the gut and the central nervous system via afferent and efferent fibers (3).

As a vital component of the parasympathetic nervous system, the vagus nerve serves as an essential bridge connecting the brain and peripheral organs, and exerts pivotal regulatory effects on memory, emotion and pain perception (4). Structurally, approximately 80% of vagal fibers are afferent fibers, while the remaining 20% are efferent fibers. The majority of afferent fibers consist of Aδ and C fibers, which transmit visceral sensory signals to the brain. By contrast, efferent fibers convey motor signals to modulate the function of peripheral organs.

Vagal efferent fibers possess prominent anti-inflammatory capacity and modulate inflammatory responses via crosstalk with the intestinal barrier and spleen, predominantly through the cholinergic anti-inflammatory pathway (CAIP). Specifically, efferent fibers release acetylcholine, which binds to receptors on splenic macrophages and suppresses the secretion of pro-inflammatory cytokines, including TNF-α, IL-1 and IL-6. In addition, the vagus nerve maintains intestinal microbiota homeostasis by regulating gastrointestinal motility and secretion, which is critical for preventing the translocation of pathogens and endotoxins into the bloodstream.

Accordingly, vagus nerve stimulation has been adopted for the management of chronic inflammatory bowel diseases such as Crohn's disease and ulcerative colitis (5). Moreover, vagus nerve stimulation yields favorable therapeutic outcomes in a spectrum of cerebral disorders, including refractory epilepsy, depression, cluster headache and migraine. It has therefore emerged as a promising non-invasive neuromodulation modality for neurological and psychiatric disorders.

Vagus nerve stimulation (VNS) can be categorized into invasive and non-invasive modalities according to delivery approaches. Invasive vagus nerve stimulation (iVNS) has proven its neuromodulatory efficacy in diseases such as epilepsy and depression. The United States Food and Drug Administration (FDA) has approved iVNS for the treatment of refractory epilepsy, major depressive disorder, obesity, as well as moderate-to-severe upper-extremity motor dysfunction following chronic ischemic stroke. Nevertheless, iVNS is associated with inherent invasiveness, high economic costs, frequent adverse reactions, and poor timeliness for acute stroke management. Its implantation procedure demands general anesthesia, which carries potential risks of complications including arrhythmia, peritracheal hematoma, and vocal cord dysfunction (6, 7), which restrict its widespread application to some extent.

In contrast, non-invasive vagus nerve stimulation is achieved via transcutaneous stimulation of auricular or cervical vagal branches (8). Among these approaches, transcutaneous auricular vagus nerve stimulation (taVNS) has attracted considerable attention because it targets the cutaneous receptive field of the auricular branch of the vagus nerve. Since taVNS does not require surgery, it avoids the potential risks associated with surgical implantation for invasive vagus nerve stimulation. Therefore, it is more suitable for stroke patients who are ineligible for surgery due to disability, anticoagulant therapy, or cardiopulmonary comorbidities, and can serve as a complementary strategy to invasive VNS. Nevertheless, current evidence remains insufficient to confirm that its efficacy is superior to implanted VNS, while it possesses broad-ranging promotion potential in clinical practice (9). TaVNS stimulates the auricular branch of the vagus nerve. After afferent signals reach the nucleus tractus solitarius, central-nervous-system regulatory effects are mediated through direct ascending and descending central pathways. In addition, taVNS can achieve multi-organ modulation via the brain-gut-immune axis, which serves as an important auxiliary mechanism for vagus nerve-mediated neuromodulation.

Given the aforementioned limitations of iVNS, the high anatomical and neurophysiological similarities between taVNS and iVNS (10), plus the inherent strengths of taVNS such as favorable safety profile, portability and low cost, transcutaneous auricular vagus nerve stimulation (taVNS) has been widely investigated as an optimal non-invasive neuromodulation technique.

This review aims to systematically summarize the mechanisms underlying taVNS, its safety profiles, and the challenges restricting its future translation into clinical practice.

## 2 Anatomy and developmental origin of the auricular vagus nerve

The vagus nerve originates from the lateral medulla oblongata. After the rootlets converge to form the main trunk, the vagus nerve exits the cranial cavity through the jugular foramen together with the glossopharyngeal nerve (cranial nerve IX) and accessory nerve (cranial nerve XI), and divides into left and right trunks. The left and right vagus nerves each enter the carotid sheath and course posterior to the common carotid artery and medial to the internal jugular vein. Their trajectories diverge upon entering the thoracic cavity:

a. The right vagus nerve crosses anterior to the right subclavian artery, passes behind the superior vena cava, and descends along the posterior aspect of the right main bronchus. It contributes fibers to the cardiac, pulmonary and esophageal plexuses. At the distal esophagus, it forms the posterior vagal trunk and finally enters the abdominal cavity via the esophageal hiatus.
b. The left vagus nerve enters the thorax between the left common carotid artery and left subclavian artery, descends alongside the aortic arch, and runs posterior to the left main bronchus. After participating in the formation of the cardiac, pulmonary and esophageal plexuses, it continues as the anterior vagal trunk and also traverses the esophageal hiatus to reach the abdominal cavity.

Upon entering the abdomen, the vagal trunks emit multiple branches and form plexuses that innervate abdominal viscera up to the distal one-third of the transverse colon.

### 2.1 Major branches and physiological functions

The vagus nerve gives off several vital branches along its course, including the pharyngeal branches, superior laryngeal nerve, and recurrent laryngeal nerve. The superior laryngeal nerve is divided into a sensory internal branch and a motor external branch. The recurrent laryngeal nerve follows a distinctive path: it curves around large thoracic vessels before ascending back to the larynx.

a. The left recurrent laryngeal nerve loops under the aortic arch at the ligamentum arteriosum and ascends within the tracheoesophageal groove.
b. The right recurrent laryngeal nerve winds around the right subclavian artery and then courses back to the neck.

Both nerves penetrate the larynx posterior to the thyroid gland and collectively regulate swallowing and phonation. In terms of sensory innervation, the internal branch of the superior laryngeal nerve supplies structures superior to the vocal folds (e.g., the epiglottis and laryngeal inlet mucosa), whereas sensation inferior to the vocal folds is conveyed by the recurrent laryngeal nerve.

### 2.2 Fiber composition and neural pathways of the vagus nerve

The vagus nerve is a mixed nerve comprising three categories of nerve fibers:

a. Motor fibers
  These fibers originate from the cerebral cortex, whose axons descend to the nucleus ambiguus in the medulla oblongata and form synapses with lower motor neurons.
b. Sensory fibers
• Somatic sensory fibers
  They transmit general somatic sensations from the laryngeal mucosa, dura mater of the posterior cranial fossa, partial skin of the external ear, as well as the posterior inferior external auditory canal and tympanic membrane. The cell bodies of primary neurons reside in the inferior vagal ganglion (laryngeal sensation) and superior vagal ganglion (auricular and dural sensation), respectively. These fibers ultimately project to the spinal trigeminal nucleus and synapse with secondary neurons.
•Visceral sensory fibers
  They relay sensory signals from the laryngopharynx, larynx, aortic bodies, and visceral organs within the thoracic and abdominal cavities. Primary neuron cell bodies are located in the inferior vagal ganglion; these fibers project to the nucleus tractus solitarius for synaptic relay.
•Special sensory (gustatory) fibers
  These fibers transmit gustatory signals from the epiglottis and laryngopharynx. Primary neuron cell bodies lie within the inferior vagal ganglion. The fibers course along the tractus solitarius and terminate in the gustatory subnucleus of the nucleus tractus solitarius.

c. Parasympathetic fibers
  Classified as visceral motor fibers, they innervate the tracheobronchial tree, esophagus, heart, and digestive tract extending to the distal transverse colon. Their preganglionic fibers arise from the dorsal motor nucleus of the vagus, descend within the vagus nerve, and synapse in ganglia situated within or adjacent to thoracic and abdominal target organs. The postganglionic fibers subsequently innervate the corresponding viscera.

### 2.3 Vagal innervation of the external ear

Sensory innervation of the external ear is intricate, primarily involving the auricular branch of the vagus nerve (ABVN), the auriculotemporal branch of the trigeminal nerve, and the great auricular nerve derived from the cervical plexus. The auriculotemporal nerve connects with the facial nerve and the otic ganglion, and receives parasympathetic fibers from the glossopharyngeal nerve before innervating the parotid gland. The great auricular nerve arises from the C2 and C3 spinal nerves; additionally, the lesser occipital nerve (C2, C3) may contribute partial cutaneous innervation. These nerves form abundant communicating branches and overlapping plexiform networks within the auricle, such as the conchal plexus and triangular fossa plexus, indicating that the functional effects of auricular acupoints rely on the synergistic activity of the entire neural network.

The auricular branch of the vagus nerve (ABVN) originates from the jugular ganglion. It merges with branches of the glossopharyngeal nerve to form the auricular branch, joins the main trunk of the facial nerve at the stylomastoid foramen, separates after exiting the tympanomastoid fissure, and ascends along the retroauricular sulcus, emitting two perforating branches. The anterior perforating branch supplies the cavum conchae, posterior auricular muscle, and superior-middle posterior auricular surface, extending to the external auditory canal, root of the helix crus, triangular fossa, antihelix, and mid-scapha. ABVN fibers are densely concentrated in the cavum conchae and cymba conchae adjacent to the origin of the helix crus.

### 2.4 Embryonic derivation of the auricular branch of the vagus nerve (ABVN)

From an embryonic developmental perspective, the auricular branch of the vagus nerve (ABVN) is a derivative nerve that originally innervates the first pharyngeal arch. The adult external auditory canal, middle ear and inner ear are residual structures derived from this arch. The ABVN arises from the superior ganglion of the vagus nerve (jugular ganglion). After fusing with the auricular branch from the inferior ganglion of the glossopharyngeal nerve, it passes through the dilated segment of the internal jugular vein and enters the mastoid canaliculus within the petrous part of the temporal bone. The superior vagal ganglion lies within the jugular foramen with an approximate diameter of 4 mm, and forms communicating rami with the petrous ganglion of the glossopharyngeal nerve, cranial root of the accessory nerve, and superior cervical sympathetic ganglion.

Approximately 4 mm above the stylomastoid foramen, the ABVN penetrates the facial nerve sulcus and joins the facial nerve via its ascending branch, which explains why herpes zoster may involve its cutaneous innervation territory. Subsequently, the nerve traverses the tympanomastoid fissure (also termed the mastoid foramen) and receives collateral branches from the glossopharyngeal nerve and cervical sympathetic trunk. Finally, it divides into two rami posterior to the auricle: one ramus anastomoses with the posterior auricular branch of the facial nerve, whereas the other supplies the auricle, posteromedial skin of the external auditory canal, and the corresponding region of the tympanic membrane.

In summary, the auricular branch of the vagus nerve (ABVN) is predominantly distributed in the cymba conchae and cavum conchae adjacent to the root of the helix crus. Except for the cymba conchae, which is fully innervated by ABVN, other auricular regions receive partial ABVN supply to varying degrees. A human cadaveric anatomical study involving seven specimens demonstrated that the proportions of ABVN innervation in the helix, cavum conchae, tragus, antihelix crus and helix crus were 73%, 45%, 45%, 20% and 9%, respectively (11). Among all auricular subregions, the concha and tragus exhibit the densest ABVN fiber projections.

## 3 Neural pathways and core mechanisms underlying taVNS

### 3.1 Afferent pathways and central roles of the nucleus tractus solitarius (NTS)

The nucleus tractus solitarius (NTS) serves as the primary termination nucleus for vagal afferent fibers and exerts a pivotal function in receiving and relaying sensory signals originating from the external ear and other peripheral regions. Afferent signals conveyed by the vagus nerve first arrive at the jugular ganglion, where the neuronal cell bodies reside, before projecting to the medullary NTS for primary signal integration.

Via direct and indirect ascending projections, the NTS forms extensive connections with multiple brain regions including the parabrachial nucleus, dorsal raphe nucleus, locus coeruleus, hypothalamus, thalamus, amygdala, and hippocampus (12–14). This broad ascending projection network enables the NTS to modulate autonomic nervous activity, emotional processing, and cognitive responses. For instance, serotonergic vagal afferent signals are integrated within the NTS, and norepinephrine released from the locus coeruleus interacts with the serotonergic system to jointly regulate mood. Furthermore, central connections formed by vagal afferent fibers innervating the lungs, heart, kidneys and other visceral organs facilitate precise coordination of physiological processes such as respiration, heart rate, and blood pressure.

### 3.2 Efferent pathways and the dorsal motor nucleus of the vagus (DMV)

The dorsal motor nucleus of the vagus (DMV) constitutes the primary origin of parasympathetic efferent vagal fibers. Preganglionic fibers arising from the DMV extensively innervate multiple thoracic and abdominal viscera, including the stomach, bronchi, small intestine, and pancreas. These fibers play a critical regulatory role in modulating gastrointestinal smooth muscle contraction, glandular secretion, and intestinal motility.

### 3.3 Afferent-efferent interactions and integrated physiological regulation

The afferent and efferent pathways of the vagus nerve form an efficient bidirectional communication system that maintains dynamic homeostasis of the autonomic nervous system through constant brain-visceral signaling crosstalk. For example, gastrointestinal sensory signals are transmitted upward via afferent fibers to the brainstem and limbic system; subsequently, parasympathetic signals are dispatched through efferent fibers to precisely modulate physiological processes such as intestinal peristalsis and digestive juice secretion. Notably, this bidirectional regulatory mechanism is not limited to the cardiovascular, respiratory and digestive systems. It also broadly participates in the modulation of immune responses, inflammatory control, emotional processing and cognitive function, and plays a vital role in sustaining bodily homeostasis and regulating a variety of physiological, neurological and psychiatric disorders (15).

### 3.4 Vagus nerve stimulation and the cholinergic anti-inflammatory pathway (CAIP)

Accumulating evidence demonstrates that transcutaneous auricular vagus nerve stimulation (taVNS) activates the vagus-NTS pathway and the cholinergic anti-inflammatory pathway, thereby exerting anti-inflammatory and analgesic effects (1, 16). After the vagus nerve detects systemic inflammatory signals, its efferent fibers are activated. The nerve terminals release acetylcholine, which binds to α7 nicotinic acetylcholine receptors (α7nAChRs) expressed on immune cells including macrophages and dendritic cells. This binding robustly blocks the secretion of pro-inflammatory cytokines such as tumor necrosis factor-α (TNF-α), interleukin-6(IL-6), interleukin-1β(IL-1β) and interleukin-8 (IL-8) (17, 18), ultimately suppressing systemic inflammatory responses.

## 4 Schematic overview of core mechanisms and clinical applications of taVNS

To systematically integrate the complete cascade of transcutaneous auricular vagus nerve stimulation (taVNS) from auricular stimulation targets to central brain nuclei and subsequent systemic effects, as well as to clarify its correlations with clinical applications, this review summarizes and presents a schematic diagram illustrating its core anatomical structures and neural pathways (Figure 1), together with a summary table of clinical applications and stimulation parameters (Figure 2).

**Figure 1:**
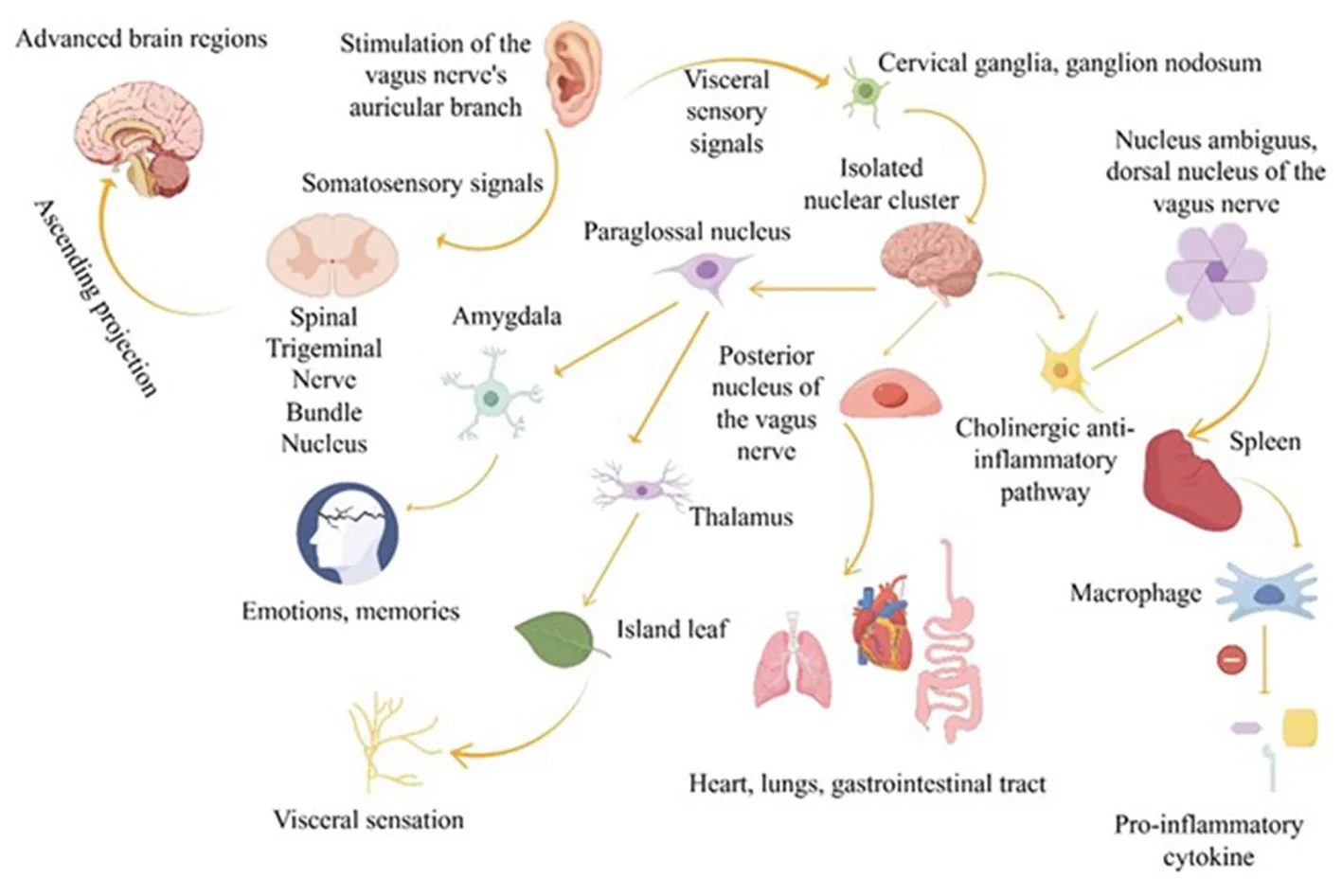
Anatomy and neural pathway diagram of taVNS.

**Figure 2:**
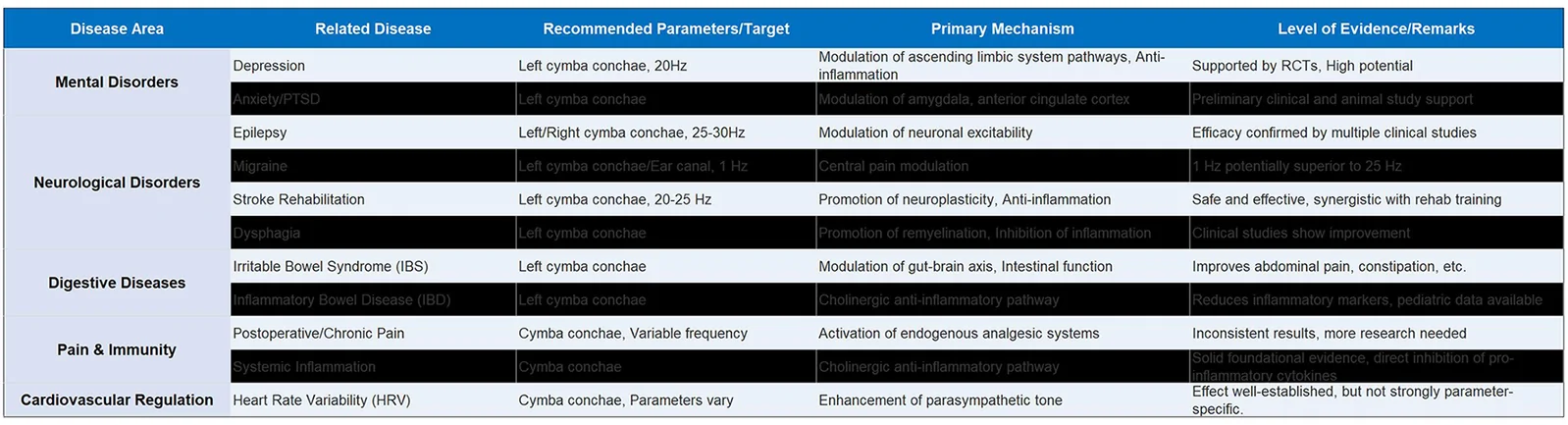
Summary of stimulation parameters reported in published taVNS studies.

As depicted in Figure 1, stimulation signals induced by taVNS originate from the auricular branch of the vagus nerve (ABVN) distributed in regions such as the cymba conchae. After these afferent signals relay to the nucleus tractus solitarius (NTS), the critical brainstem hub, taVNS exerts regulatory effects on emotional and cognitive functions, autonomic activity, and systemic inflammatory status via ascending, descending, and neuroimmune (cholinergic anti-inflammatory) pathways, respectively.

On the basis of this multi-dimensional mechanistic framework, Figure 2 further links the above neural pathways to specific disorders and systematically sorts out potential stimulation targets and parameter settings for epilepsy, depression, pain disorders, gastrointestinal diseases and other conditions. This figure summarizes stimulation parameters reported in published taVNS studies for descriptive purposes only and does not constitute clinical recommendations. Heterogeneity regarding study design, sample size, comparator conditions, outcomes and evidence levels of relevant taVNS trials are described in the main text. These findings reveal the great translational potential and inherent complexity of taVNS as a cross-disease neuromodulation technique.

## 5 Technical parameters and stimulation protocols

With the advancement of vagus nerve stimulation technology, transcutaneous auricular vagus nerve stimulation (taVNS) devices have undergone continuous innovation. Based on current classification criteria, such devices are divided into three main categories. First, conventional transcutaneous electrical nerve stimulation (TENS) devices are limited in clinical application due to their bulky size and poor electrode compatibility. Second, specialized miniaturized devices exclusively developed to target the auricular branch of the vagus nerve (ABVN) enable non-invasive or minimally invasive stimulation of multiple auricular sites including the concha and external auditory canal. A representative product, TENS-200, achieves optimized electrode design and reduced device volume. Third, integrated, intelligent and miniaturized devices (e.g., VITALITY Smart Cable™) feature a higher level of technical integration.

Despite continuous upgrades of stimulation devices, no consensus has been reached regarding the optimal therapeutic protocol for taVNS. In terms of stimulation targets, although the quantity of Aβ fibers within the left and right auricular branch of the vagus nerve (ABVN) is comparable, it has long been recognized that vagal fibers associated with cardiac regulation are predominantly distributed on the right side. For safety concerns, most existing studies adopt left auricular stimulation. To date, no studies have clarified the safety profile of right-sided taVNS, and this issue remains to be further verified.

The configuration of stimulation parameters also presents considerable complexity. There exist diverse combinations of pulse width, frequency, and intensity, and the therapeutic outcomes may vary across distinct disease entities. Current clinical investigations of taVNS cover a wide spectrum of disorders, mainly including epilepsy, depression, insomnia, pain, stroke, and post-stroke sequelae.

a. Pulse width: Clinical studies adopt a wide range of pulse widths, with commonly used values including 100 μs, 200–300 μs, 450 μs, and even 1000 μs. Some literatures report the routine use of 1 ms or 0.25 ms (ranging from 0.02 to 1 ms) (19), other evidence suggests that a pulse width of 500 μs yields the strongest biological activity (20).
b. The intensity settings vary dramatically, ranging from sub-milliamperes (e.g., 0.5 mA) to tens of milliamperes (e.g., 10–50 mA). Several studies set the intensity based on participants' tingling threshold, pain threshold, or maximum tolerable threshold. Given that most trials fail to report detailed intensity protocols and often allow self-adjustment by subjects, the optimal stimulation intensity remains undefined to date.
c. Stimulation frequency: This parameter has been relatively extensively investigated. Frequencies commonly applied include 1 Hz, 20 Hz, 25 Hz, and 30 Hz. Distinct therapeutic effects have been observed across different frequencies. In rodent models of depression, 20 Hz stimulation produces superior antidepressant-like effects (21). A human randomized double-blind trial demonstrated that high-frequency stimulation at 25 Hz enhances gastric antral motility more prominently than low-frequency 1 Hz stimulation (22). Nevertheless, the therapeutic benefit does not always correlate positively with stimulation frequency. For patients with chronic migraine, taVNS at 1 Hz reduces headache days more effectively than 25 Hz (23), a finding further validated by subsequent animal experiments. Low-frequency stimulation (15 Hz) exerts stronger inhibitory effects on serum TNF-α and IL-1β levels compared with high-frequency stimulation (25 Hz), and this anti-inflammatory effect is independent of stimulation duration (24).

Parameter-dependent therapeutic effects may also differ according to stimulation targets and laterality. The cavum conchae and triangular fossa exert prominent regulatory effects on heart rate variability (HRV), with distinct left-right auricular differences. Stimulation intensity may exert a greater impact on autonomic responses than stimulation frequency. Under certain conditions, 80 Hz stimulation delivered to the external auditory canal yields superior outcomes compared with 25 Hz stimulation applied to the auricular concha.

In summary, unified standards for taVNS have not yet been established regarding device selection, stimulation targets, and parameter configuration. High-quality large-scale clinical trials targeting specific diseases are required in future research to develop individualized optimized therapeutic protocols.

## 6 Comparison between invasive VNS and taVNS

Invasive VNS surgically places electrodes around the cervical vagus nerve trunk, providing well-defined stimulation targets and a wide adjustable range of stimulation parameters, with abundant high-quality clinical evidence accumulated. However, invasive VNS requires surgical implantation, bringing procedure-related risks such as infection, vocal cord palsy and vascular injury, together with high medical costs and limited flexibility for post-implantation parameter adjustment. By contrast, taVNS targets the auricular branch of the vagus nerve without surgical intervention. It exhibits favorable safety with mild adverse events, low treatment cost, and allows repeated intermittent stimulation with high patient acceptance. Nevertheless, taVNS has notable limitations: it delivers indirect nerve stimulation with relatively low target precision, considerable inter-individual response variability, and lacks standardized stimulation protocols. Overall, the level of clinical evidence for taVNS is inferior to that of invasive VNS. Instead of being mutually substitutable, these two modalities are complementary and suitable for distinct clinical scenarios and populations. Relevant review regarding vagus nerve-mediated brain modulation and post-stroke remodeling is cited herein (25).

## 7 Clinical applications and therapeutic evidence

### 7.1 Psychiatric disorders

Depression is closely associated with autonomic nervous system imbalance, which may arise from dysfunction of multiple brain regions involved in emotional and cognitive regulation, including the hippocampus (HIP), anterior cingulate cortex (ACC), and medial prefrontal cortex (MPFC). These brain regions are modulated by the microbiome-gut-brain axis. Core depressive symptoms consist of persistent low mood, anhedonia, and altered sleep and appetite, which may be accompanied by cognitive impairment or psychotic manifestations (26, 27). Depression represents a major risk factor for suicide across all age groups. Current therapeutic strategies for depression mainly include pharmacotherapy, psychotherapy, cognitive behavioral therapy, deep brain stimulation, electroconvulsive therapy, and repetitive transcranial magnetic stimulation. Although antidepressants serve as first-line treatment, their overall efficacy is modest, accompanied by risks of disease recurrence and drug resistance. Other interventions are also limited in widespread clinical application for depression due to high costs, equipment barriers, and poor patient tolerability.

Since the publication of the first clinical trial validating the antidepressant efficacy of vagus nerve stimulation in 2013, a growing body of studies has corroborated its anti-depressive effects. While invasive vagus nerve stimulation exerts anti-inflammatory effects, its potential adverse events such as surgical complications restrict its clinical adoption. In contrast, transcutaneous auricular vagus nerve stimulation (taVNS) is an emerging non-invasive neuromodulation technique with favorable safety and tolerability profiles. Mechanistic investigations have demonstrated that taVNS alleviates depressive and anxiety-like behaviors in adolescents by regulating hippocampal glycolysis and inflammatory responses, and relieves depressive-like phenotypes in rodent models via modulating peripheral-central inflammatory pathways (28). Meta-analyses have reported elevated levels of pro-inflammatory cytokines including tumor necrosis factor-α, interleukin-6, interleukin-1, and C-reactive protein during depressive episodes (29), indicating that taVNS ameliorates depressive symptoms through suppressing inflammatory responses.

TaVNS also yields promising therapeutic effects for anxiety and related disorders. One randomized controlled trial confirmed that taVNS effectively relieved anxiety symptoms among retired teachers (30). Post-traumatic stress disorder (PTSD) is a debilitating neuropsychiatric disorder triggered by traumatic events, frequently complicated by severe anxiety. Preclinical animal studies have verified that vagus nerve stimulation alleviates PTSD-like symptoms in rodents (31). Further mechanistic research revealed that taVNS mitigates anxiety-like behaviors in PTSD mice by modulating glutamatergic neurons within the anterior cingulate cortex (32).

In conclusion, taVNS stands as a promising therapeutic modality for psychiatric illnesses including depression and anxiety. Nevertheless, existing research within this field is largely confined to animal models and small-sample clinical trials; future large-scale clinical investigations are urgently warranted.

### 7.2 Neurological disorders

Epilepsy is a chronic central nervous system disorder characterized by recurrent seizures. Transcutaneous auricular vagus nerve stimulation (taVNS) was first adopted for epilepsy management, and substantial evidence supporting its anti-epileptic effects has been accumulated to date. Early small-cohort studies demonstrated that taVNS safely reduces seizure frequency (33, 34), while subsequent large-scale investigations and randomized controlled trials further validated its therapeutic efficacy and safety for epilepsy treatment (35, 36). In addition, vagus nerve stimulation can partially improve verbal recognition memory in patients with epilepsy.

Migraine is another neurological disorder manifested as severe throbbing headache, frequently accompanied by photophobia and phonophobia. It impairs executive function, memory and attention, thereby severely compromising patients' quality of life (37–40). TaVNS has exhibited promising efficacy for migraine in patients aged 27–29 years (41), effectively shortening headache duration and lowering seizure frequency and intensity. Notably, stimulation frequency is a critical parameter for migraine management. Multiple studies have confirmed that 1 Hz taVNS outperforms 25 Hz in reducing monthly headache days (23, 42, 43), which provides important guidance for parameter optimization in future taVNS clinical practice.

Stroke ranks among the leading global causes of adult mortality and disability. Many stroke survivors still suffer persistent impairments in motor, sensory, coordination, language, visual and cognitive domains despite standard rehabilitation training (44). Studies have illustrated that invasive vagus nerve stimulation (iVNS) combined with rehabilitation training improves upper limb motor weakness in patients with chronic ischemic stroke (45). However, its invasive nature and surgical risks create an urgent demand for non-invasive alternatives. Featuring favorable safety, satisfactory tolerability and overlapping neural pathways with iVNS, taVNS represents an appealing therapeutic strategy for stroke rehabilitation. One randomized controlled trial enrolling stroke patients revealed that taVNS plus conventional rehabilitation yielded significantly greater improvements in motor function, sensory performance and emotional status compared with sham stimulation combined with routine rehabilitation, without obvious adverse events (46). Emerging clinical data suggest that taVNS may relieve motor-related symptoms in Parkinson's disease. Mechanistically, non-invasive vagus nerve stimulation could reshape cerebral cortical network topology and facilitate intracortical facilitation, providing a potential cortical-level explanation for motor improvement (47). Apart from cortical functional reorganization, non-invasive vagus-nerve stimulation also yields measurable clinical benefits accompanied by peripheral and central molecular alterations in Parkinson's disease (48). These findings highlight multi-level mechanisms underlying vagus neuromodulation and offer new biomarkers for future taVNS trials. Mechanistically, taVNS exerts therapeutic benefits by boosting activation of injured brain regions and facilitating neuroplasticity (1, 49), involving remyelination, angiogenesis and anti-inflammatory responses. Most existing studies concerning taVNS for stroke focus on ischemic stroke. Notably, hemorrhagic stroke represents another highly disabling stroke subtype. Recent pre-clinical animal studies have indicated that taVNS may facilitate structural and functional remodeling of sensorimotor networks after intracerebral hemorrhage (50). Nevertheless, these encouraging findings are limited to animal models; adequate clinical evidence in patients with hemorrhagic stroke is still absent and awaits further investigation. Furthermore, taVNS produces favorable outcomes for post-stroke dysphagia (51). Cognitive dysfunction is a prevalent post-stroke complication, yet research exploring taVNS effects on post-stroke cognitive impairment remains in preliminary stages; its optimal stimulation parameters and overall therapeutic effects require further investigation.

### 7.3 Other therapeutic fields

#### 7.3.1 Inflammatory bowel disease and irritable bowel syndrome

Irritable bowel syndrome (IBS) is a common functional gastrointestinal disorder characterized by abdominal pain and altered bowel habits. Although IBS is not life-threatening, it substantially impairs patients' quality of life and increases socioeconomic and healthcare burdens (52). The vagus nerve exerts potent anti-inflammatory properties and may relieve abdominal pain and constipation in IBS patients by modulating neuroimmune responses, intestinal microbiota, and interstitial cells of Cajal (53, 54). The management of inflammatory bowel disease (IBD) remains challenging due to incomplete therapeutic responses, adverse drug reactions, and high treatment costs. Clinical trials have demonstrated that taVNS reduces fecal calprotectin levels and alleviates clinical symptoms in pediatric patients with mild-to-moderate IBD (55, 56), providing novel evidence for the therapeutic application of taVNS in IBD treatment. Currently, standardized optimal treatments are lacking for both IBS and IBD, and taVNS has emerged as a promising alternative therapeutic strategy. Accumulating studies have validated that taVNS effectively improves core symptoms including abdominal pain and constipation; however, further high-quality investigations are required to establish optimal stimulation parameters and maximize clinical therapeutic efficacy.

#### 7.3.2 Pain management

Pain constitutes a critical factor affecting the quality of postoperative recovery. As a potent stressor, pain triggers activation of the hypothalamic-pituitary-adrenal (HPA) axis, which elevates the secretion of stress hormones and metabolic rate while suppressing anabolism, thereby exerting varying impacts on endocrine function, immune responses and blood circulation. Inadequate pain control may induce severe anxiety, depression and impaired sleep quality, hinder postoperative rehabilitation and reduce patient satisfaction. Clinicians commonly alleviate postoperative pain via oral or intravenous analgesics, patient-controlled intravenous analgesia, regional nerve blocks and local anesthetic infiltration. Nevertheless, these interventions carry risks of adverse reactions including respiratory depression, nausea, vomiting, hyperalgesia and restricted early mobilization; additionally, opioid analgesics possess potential addictive properties.

Recent studies have revealed that taVNS relieves pain via multiple mechanisms, such as anti-inflammatory effects, modulation of the locus coeruleus-noradrenergic signaling pathway, and activation of serotonergic and endorphinergic systems (57–59). Moreover, taVNS exhibits analgesic potential in patients with systemic lupus erythematosus (SLE) (60). Although existing studies report inconsistent findings regarding the effect of taVNS on opioid consumption, taVNS can mitigate acute pain and reduce the incidence of chronic pain.

#### 7.3.3 Cardiac autonomic regulation

The autonomic nervous system is essential for maintaining cardiovascular homeostasis. Excessive sympathetic activation during heart failure or physiological aging exerts detrimental effects on cardiovascular function. Furthermore, reduced cardiac vagal activity is closely associated with cardiovascular morbidity and mortality (61). As a core component of the autonomic nervous system, the vagus nerve serves as a key regulatory target; accumulating evidence indicates that vagus nerve stimulation modulates autonomic tone and further regulates cardiovascular function (62). Enhanced vagal tone reduces cardiovascular risk factors in both animal models and human subjects, suppresses or terminates ventricular arrhythmias induced by myocardial ischemia, and elevates the ventricular fibrillation threshold (63–65). Yu et al. demonstrated that chronic low-intensity taVNS attenuates ventricular arrhythmias, inhibits left stellate ganglion activity, and ameliorates sympathetic remodeling in canine models of myocardial infarction (66). Beyond brain-gut axis regulation, taVNS modulates autonomic nervous activity indexed by heart-rate variability. Recent work on non-invasive vagus nerve stimulation further supports the close interplay between cortical excitability and autonomic modulation following neuromodulation (67). Such combined cortical-autonomic changes may partially explain the broad systemic effects induced by vagus nerve stimulation. Heart rate variability (HRV), defined as the physiological fluctuation in consecutive heartbeat intervals, serves as a reliable indicator of multifaceted cardiac regulatory mechanisms and a non-invasive biomarker for evaluating cardiac autonomic function, reflecting efferent vagal activity (68). Reduced HRV is strongly correlated with increased morbidity and mortality across various diseases, particularly cardiovascular disorders.

Clancy et al. verified that taVNS improves HRV and suppresses sympathetic activity by modulating the low-frequency to high-frequency power ratio, demonstrating its capacity to restore cardiac autonomic balance in healthy individuals (69). Recent studies further confirm that taVNS effectively elevates HRV, which may provide therapeutic benefits for HRV-deficient disorders, though such effects appear to be non-parameter-specific (70). As cardiovascular diseases pose severe threats to human health, the therapeutic potential of taVNS in cardiovascular autonomic modulation has been preliminarily validated. Further rigorous investigations are warranted to explore its clinical application value and underlying mechanisms.

## 8 Safety and adverse reactions

TaVNS generally exhibits a favorable safety profile. Mild auricular pain, headache or tingling sensations at the stimulation site are the most common adverse effects (71), most of which correlate with stimulation duration. Transient reflexive responses mediated by vagal reflexes may also occur, including coughing, throat tightness, voice alteration, facial muscle twitching, nausea and gastrointestinal discomfort.

All adverse reactions reported for vagus nerve stimulation to date are mild. Data regarding taVNS application remain scarce for patients with implanted cardiac devices, severe bradyarrhythmias, or cutaneous lesions around the auricle. Therefore, clinical administration requires caution in individuals with severe arrhythmias, active ulcers, uncontrolled asthma, carotid sinus syndrome, or bilateral vagal nerve injury (72). Rigorous risk-benefit assessment is also mandatory prior to taVNS use in special populations such as pregnant women, children, and patients with cardiac pacemakers.

## 9 Current challenges and future perspectives

Current research on taVNS is confronted with several core challenges. First of all, stimulation parameters have not been standardized. Protocols vary drastically across studies in terms of frequency, intensity and treatment duration, which obscures the dose-effect relationship. Such heterogeneity impedes cross-study comparison and result reproducibility, and hinders the optimization of therapeutic efficacy.

Second, the underlying mechanisms remain to be fully elucidated. Although animal experiments have provided reasonable mechanistic explanations for vagal modulation, the precise neural pathways and molecular mechanisms are not yet comprehensively understood. Moreover, translational findings need to be validated in large-scale human clinical cohorts to generate meaningful clinical outcomes.

Third, the overall level of clinical evidence requires improvement. Most existing trials are limited by small sample sizes and insufficient long-term follow-up data; large-scale, high-quality investigations are still needed to confirm the long-term safety and sustained therapeutic effects of taVNS. In addition, current studies mainly enroll relatively healthy adult subjects, while evidence regarding high-risk populations (e.g., patients with severe cardiopulmonary diseases) remains scarce.

## 10 Discussion

This narrative review demonstrates that transcutaneous auricular vagus nerve stimulation (taVNS), a non-invasive neuromodulation technique, exerts therapeutic potential via multi-modal regulation of the brain-gut-immune axis. By stimulating the auricular branch of the vagus nerve, taVNS transmits afferent signals to the nucleus tractus solitarius (NTS), the central integrative hub. Subsequent ascending pathways modulate emotional and cognitive functions, while descending pathways maintain autonomic homeostasis. Critically, taVNS robustly activates the cholinergic anti-inflammatory pathway to suppress the release of pivotal pro-inflammatory cytokines including TNF-α and IL-6. This multi-target mechanistic framework lays a solid theoretical foundation for taVNS applications across psychiatric, neurological, gastrointestinal and other disorders, with preliminary therapeutic efficacy validated by clinical investigations.

Despite promising prospects, translational implementation of taVNS from preclinical laboratories to routine clinical practice faces fundamental obstacles. The most prominent bottleneck lies in the substantial lack of standardized stimulation protocols, encompassing stimulation laterality (debates over safety and efficacy discrepancies between left and right auricles), pulse width, intensity, and particularly frequency selection. For instance, low-frequency protocols (1 Hz or 20 Hz) yield superior outcomes for migraine and depression, whereas high-frequency stimulation (25 Hz) demonstrates greater efficacy in enhancing gastric motility. Such heterogeneous parameter-dependent effects indicate that no universal “one-size-fits-all” stimulation protocol exists for taVNS. Therapeutic responses are jointly determined by disease entity, individual physiological status, and stimulation location. Future research should prioritize developing disease-specific parameter frameworks tailored to distinct pathophysiological mechanisms, rather than pursuing a universal stimulation regimen.

With regard to safety, cumulative evidence consistently confirms the favorable safety and tolerability profile of taVNS, with predominant adverse events confined to mild, transient discomfort at stimulation sites. Nevertheless, risks associated with taVNS administration require further evaluation through large-scale long-term trials for vulnerable populations, including patients with severe arrhythmias or uncontrolled asthma, as well as pregnant women and children. Individualized protocol adjustment constitutes the core guarantee of patient safety and comfort.

Looking ahead, research within the taVNS field should focus on the following directions. First, well-designed large-scale, multicenter randomized controlled trials are required to define optimal stimulation parameters for specific diseases and resolve ambiguous dose-effect relationships. Second, advanced neuroimaging modalities such as functional magnetic resonance imaging (fMRI) and high-density electroencephalography, combined with humoral biomarker profiling, will facilitate comprehensive elucidation of precise human *in-vivo* signaling pathways and identification of predictive biomarkers for therapeutic responsiveness. Furthermore, technological integration and individualized treatment represent an inevitable developmental trend. This includes the development of home-use taVNS devices integrated with wearable sensors and remote monitoring systems, alongside machine learning models that predict personalized optimal parameters based on physiological signals such as heart rate variability (HRV), ultimately realizing precise neuromodulation. Finally, further exploration of novel clinical indications including perioperative management and neurodegenerative diseases, as well as combinatorial regimens with conventional therapies (pharmacotherapy, psychotherapy etc.), may deliver novel therapeutic alternatives for broader patient populations. Beyond established indications such as epilepsy, depression and pain, taVNS exhibits promising research potential for disorders of consciousness (DoC) rehabilitation, perioperative care, neurodegenerative diseases (e.g., Parkinson's disease), and emerging fields including motion sickness management.

### 10.1 Limitations

This review has several inherent limitations. First, the literature screening process may be biased by database sources and language restrictions, which could lead to the exclusion of certain relevant studies. Second, the quality of existing clinical trials on taVNS varies substantially. Most investigations are characterized by small sample sizes and short follow-up durations, making reliable pooled effect-size analysis difficult to conduct. Moreover, the mechanistic discussion of taVNS in this paper is primarily based on animal experiments and neurophysiological theories; additional direct human evidence is required to clarify its precise neuromodulatory pathways *in vivo*. Despite these limitations, the present review comprehensively summarizes the current research landscape of taVNS and provides references for the design of future investigations.

## 11 Conclusion

As an emerging non-invasive neuromodulation technique, transcutaneous auricular vagus nerve stimulation (taVNS) has demonstrated promising therapeutic potential across multiple clinical fields including psychiatric, neurological and gastrointestinal disorders. Although most studies have reported only mild adverse events, discomfort induced by local stimulation and vagal reflexes, as well as potential risks for special populations, cannot be overlooked. The core priority for future research is to address the challenges of standardized stimulation protocols and individualized therapeutic regimens through large-scale, high-quality clinical trials, and to further elucidate its underlying mechanisms of action.
